# Association between intestinal permeability, systemic inflammation, and response to anti-TNF therapy in patients with rheumatoid arthritis: a prospective controlled study

**DOI:** 10.3389/fimmu.2026.1756621

**Published:** 2026-03-27

**Authors:** Aimara García-Studer, Arkaitz Mucientes, Jose Manuel Lisbona-Montañez, Patricia Ruiz-Limón, Sara Manrique-Arija, Fernando Ortiz-Márquez, Christopher Hinestroza-Echavarría, Laura Cano-García, Natalia Mena-Vázquez, Antonio Fernández-Nebro

**Affiliations:** 1 Instituto de Investigación Biomédica de Málaga y Plataforma en Nanomedicina-Instituto de Investigación Biomédica de Málaga (IBIMA) Plataforma Centro Andaluz de Nanomedicina y Biotecnología (BIONAND), Málaga, Spain; 2 Unidad de Gestión Clínica (UGC) de Reumatología, Hospital Regional Universitario de Málaga, Málaga, Spain; 3 Departamento de Medicina. Universidad de Málaga, Málaga, Spain; 4 UGC de Endocrinología y Nutrición, Hospital Clínico Virgen de la Victoria, Málaga, Spain; 5 Centro de Investigación Biomédica en Red (CIBER) Fisiopatología de la Obesidad y Nutrición (CIBEROBN), Instituto de Salud Carlos III, Madrid, Spain; 6 Departamento de Microbiología, Facultad de Medicina. Universidad de Málaga, Málaga, Spain

**Keywords:** anti-TNF, inflammation, intestinal permeability, rheumatoid arthritis, tight junction proteins

## Abstract

**Objectives:**

To evaluate the association between biomarkers related to intestinal epithelial barrier integrity, systemic inflammation, and clinical response to anti-TNF therapy in patients with rheumatoid arthritis (RA).

**Methods:**

A prospective controlled 24-week study of patients with active RA receiving anti-TNF therapy was performed. Findings were compared with those of age- and sex-matched healthy controls. Values of tight junction proteins (occludin, claudin-1), zonulin, lipopolysaccharide (LPS), and LPS-binding protein (LBP) were determined in serum and feces at baseline and at 6 months. The associations with clinical, inflammatory, and remission parameters (DAS28-CRP ≤2.6) were analyzed. Multivariate models explored links between intestinal, inflammatory, and treatment response biomarkers.

**Results:**

The study population comprised 70 patients with RA and 70 controls. Baseline serum levels of occludin and claudin-1 were lower in patients than in controls (p<0.001). After 6 months, systemic inflammation had improved significantly, and values of several biomarkers had returned to normal in patients who achieved clinical remission. In multivariate analysis, higher baseline occludin and claudin-1 levels were associated with a greater probability of achieving remission (OR = 1.04, and 1.02, respectively). Average HAQ was inversely associated with remission (OR = 0.26). Increased occludin after anti-TNF was associated with baseline DAS28-CRP (β=0.314) and IL-1β (β=0.416); claudin-1 with male sex (β=–0.342); and zonulin with lower IL-1β (β=–0.313) and higher resistin (β=0.294).

**Conclusions:**

Biomarkers of intestinal integrity, especially serum occludin, are altered in patients with RA and were associated with response to anti-TNF. Disruption of the intestinal barrier, as reflected by these indirect markers, is associated with systemic inflammation, thus reinforcing the gut-joint axis as a potential therapeutic target.

## 1 Introduction

Rheumatoid arthritis (RA) is a chronic inflammatory disease that mainly affects the joints and frequently other organs. If left untreated, RA leads to joint destruction, disability, and increased mortality. Patients can be treated effectively with combinations of conventional synthetic, biologic, and targeted synthetic disease-modifying antirheumatic drugs (DMARDs) (1). Tumor necrosis factor (TNF) inhibitors (anti-TNF agents) are currently the most widely used biologics because of their satisfactory cost-benefit ratio. Nevertheless, a significant percentage of patients do not respond appropriately (1). Although factors such as sex, age, antibody levels, inflammatory markers, body mass index (BMI), and smoking influence this variability, they do not fully explain or predict therapeutic outcomes (2–8). Consequently, new pathogenic biomarkers are being actively explored.

In recent years, the hypothesis that a gut-joint axis underlies onset and progression has become increasingly relevant in the pathogenesis of RA. This hypothesis proposes that alterations in the intestinal microbiome would lead to local inflammation capable of overcoming immune tolerance and progressing systemically to affect joints and organs (9). The role of tight junction protein (TJPs) and their association with intestinal inflammation in the pathogenesis of RA has been studied. TJPs such as occludin and the claudins form supramolecular complexes together with other cytoplasmic and membrane proteins that selectively regulate intestinal permeability to antigens, toxins, and bacteria, potentially triggering local inflammation and systemic diseases (10, 11). Therefore, alterations or disruptions in these proteins can compromise intestinal barrier integrity, leading to increased permeability. TJPs are modulated by zonulin, a regulatory protein that is synthesized mainly in intestinal and hepatic cells. On its release, zonulin acts on TJPs, causing the intercellular spaces to open. For their part, lipopolysaccharides (LPS), which are essential components of the outer membrane of translocated gram-negative bacteria, also play an important role in RA by increasing inflammatory burden and favoring the onset of autoimmune processes. With respect to these molecules, it has been observed that the greater baseline bioactivity of LPS predicts greater disease activity and a lower likelihood of remission. Similarly, high levels of LPS-binding protein (LBP) at the onset of symptoms have been associated with a poorer response to treatment (12).

The few clinical studies that have evaluated TJPs specifically in patients with RA generally report altered levels or distribution associated with inflammation and the response to DMARDs; however, these findings are inconsistent across studies.

Nevertheless, findings generally support the hypothesized association between systemic inflammation and disruption of intestinal integrity and the idea that control of inflammation with anti-TNF agents could restore, at least in part, intestinal integrity and leakage. This hypothesis in turn reinforces the notion of a gut-joint axis in the pathogenesis of RA and points to its usefulness as a biomarker for diagnosis and response to treatment.

The objectives of the present study, therefore, were to evaluate changes in serum biomarkers related to intestinal epithelial barrier integrity (occludin, claudin-1, and zonulin) in patients with RA and moderate-high inflammatory activity after 6 months of anti-TNF therapy and to assess their association with systemic biomarkers of inflammation, clinical disease activity, and clinical response to treatment.

## 2 Patients and methods

### 2.1 Study design

The study was a single-center, prospective, observational, controlled cohort study performed over 24 weeks. The study population included biologic-naive RA patients who, under conditions of daily clinical practice, began their first biologic therapy with an anti-TNF agent. All the patients were evaluated at initiation of treatment (baseline visit or V0), at 3 months (V3), and at 6 months (V6), although the present analysis was limited to data from V0 and V6. Before participating in the study, all participants signed the informed consent document. The study protocol was approved by the Research Ethics Committee of Hospital Regional Universitario de Málaga (HRUM), with code PI22_01207.

### 2.2 Study population

#### 2.2.1 Patients with RA

Between June 2022 and June 2023, patients from a prospective established RA cohort were included. The patients fulfilled the following criteria (1): a diagnosis of RA according to the 2010 ACR/EULAR classification criteria (13), (2) age >16 years, and (3) initiation of the first biologic treatment with an anti-TNF agent owing to moderate-high disease activity according to the 28-joint Disease Activity Score based on CRP (DAS28-CRP), despite receiving conventional synthetic DMARDs. Patients with other rheumatic diseases, patients on extreme diets, and patients who had received antibiotics or probiotics during the previous 3 months were excluded.

#### 2.2.2 Control group

A control group was included to determine whether baseline TJP levels were altered in patients with RA. The control group comprised healthy age- and sex-matched volunteers (age >16 years) without inflammatory disease. The controls were recruited from the same social environment as the patients and were subject to the same exclusion criteria as the patients.

### 2.3 Protocol

The evaluations were performed by rheumatologists and specialized nurses following the principles of Good Clinical Practice. Sociodemographic, clinical, and laboratory data were collected using a structured protocol at each of the 3 visits. The clinical data were collected by rheumatologists.

The data recorded were as follows: tender and swollen joint counts, the patient’s and physician’s visual analog scale (VAS) score, the DAS28-CRP (14), and the Health Assessment Questionnaire (HAQ [Spanish version]) score (15). Biologic samples (blood, feces, and urine) were collected after an 8-hour fast and processed according to standard procedures before being stored at –80 °C in the biobank, thus fulfilling all quality, traceability, and coding requisites.

### 2.4 Outcome measures

#### 2.4.1 Main variables

The main dependent variable was remission at 6 months, defined as DAS28-CRP ≤2.6. Absence of remission was defined as DAS28-CRP >2.6. Patients were also classified based on the EULAR criteria (16) as good responders, moderate responders, and nonresponders according to both the decrease in DAS28-CRP and to the degree of disease activity at the end of follow-up.

In order to evaluate the net effect of anti-TNF therapy on the integrity of the epithelial barriers, the dependent variables applied were absolute changes (Δ) in the concentrations of occludin, claudin-1, and zonulin, calculated as the difference between the post-treatment and baseline values.

#### 2.4.2 Sociodemographic, anthropometric, and clinical values

The sociodemographic and anthropometric data recorded were age, sex, ethnic group, and body mass index (BMI) according to the recommendations of the World Health Organization (45). The clinical data recorded were comorbidities included in the Charlson comorbidity index (CCI) (both crude and age-adjusted [ACCI]) (17), lifestyle, substance use (smoking habits, alcohol consumption), and cardiovascular risk factors, including hypertension (18), diabetes (19), dyslipidemia (total cholesterol >200 mg/dL, low-density lipoprotein [LDL] cholesterol >160 mg/dL, or triglycerides >175 mg/dL) (20), and obesity (BMI ≥30 kg/m²) (21). History of cardiovascular disease and mood disorders according to the Hospital Anxiety and Depression Scale (HADS) were also recorded (22).

#### 2.4.3 Disease characteristics

At each visit, the disease characteristics recorded were date of onset of symptoms (in the clinical history), date of diagnosis (2010 ACR/EULAR criteria) (13), disease duration, tender and swollen joint counts, and scores for the patient and physician VAS, DAS28-CRP (14), and HAQ (15) (Spanish version). Inflammatory activity from inclusion in the cohort until the baseline visit (V0) based on the average of the DAS28-CRP values from of the previous visits was also recorded. Radiographic evidence for the presence of erosions was only recorded at baseline.

#### 2.4.4 Concomitant treatment

Therapy with synthetic DMARDs (methotrexate, leflunomide, sulfasalazine, or hydroxychloroquine) and corticosteroids was recorded. This included the dose being taken at each visit.

#### 2.4.5 Laboratory variables

Basic laboratory values and specific markers of permeability, autoimmunity, and inflammation (RF, anti–citrullinated peptide antibody [ACPA], CRP, and erythrocyte sedimentation rate [ESR]) were recorded. An analysis of more specific inflammatory mediators was performed (interleukin [IL] 1β [IL-1β], IL-6 [Q6000B, R&D Systems Inc., Minneapolis, USA], oxidized LDL [BI-20032, Biomedica GmbH, Vienna, Austria], and TNF-α [QTA00C, R&D Systems Inc., Minneapolis, USA]).

Variables associated with intestinal barrier integrity were measured in serum and feces according to the manufacturer’s instructions. The variables measured were occludin (CSB-EL016263HU, CUSABIO Innovation Center, Houston, TX, USA), claudin-1 (CSB-EL005490HU, CUSABIO), and zonulin-related proteins (CSB-EQ027649HU, CUSABIO). ELISA kits were used to measure LPS (EKC34448, Biomatik) and LBP (CSB-E09629h, CUSABIO) in serum. Serum values of adipokines (leptin, resistin, and adiponectin) and insulin-like growth factor 1 (IGF-1) were determined in patients and controls using ELISA (Mediagnost GmbH, Tübingen, Germany).

### 2.5 Statistical analysis

A descriptive analysis of the characteristics of the sample was performed. Categorical variables are expressed as frequency and percentage; quantitative variables are expressed as mean and standard deviation (SD) or median and interquartile range (IQR), depending on the normality of their distribution (Kolmogorov-Smirnov test).

The χ^2^ and *t* test or Mann-Whitney test were performed to compare the main characteristics between the study groups (patients and controls) at baseline or within the groups. Matched groups were compared during follow-up using the paired samples *t* test or the Wilcoxon test. The correlation between parameters was evaluated using the Pearson or Spearman test. Finally, multivariate models were constructed based on the main study variables. In line with international recommendations on sex and gender in biomedical research and evaluation of the biological variability associated with these variables, analyses stratified by sex were performed. These were systematically included in all the multivariate models, together with other key variables such as age and smoking, both to adjust for the effect and to explore specific interactions.

A multivariate logistic regression model (dependent variable: remission) was constructed to determine independent associations with remission at 6 months. This was followed by a multiple linear regression analysis to evaluate the association between the independent variables and the dependent variables (Δ) occludin, (Δ) claudin-1, and (Δ) zonulin after 6 months of anti-TNF therapy.

The statistical analyses were performed using IBM SPSS Statistics for Mac OS, Version 28 (IBM Corp., Armonk, NY, USA), licensed to staff at the University of Malaga. Statistical significance was set at p < 0.05.

## 3 Results

### 3.1 Patients and controls: baseline characteristics

#### 3.1.1 Baseline epidemiological, clinical, and treatment variables

The study population comprised 70 patients with RA and 70 controls. **Table 1** shows the main baseline characteristics. Most RA patients were white women (82.9%) with a mean (SD) age of 55.3 years (15.1). Inflammatory activity was moderate-high in all patients at baseline (V0), before initiation of the first biologic.

**Table 1:** Clinical and epidemiological characteristics of patients with RA and healthy controls.

Variable	RA (N = 70)	Controls (N = 70)	P value
Epidemiological			
Female sex, n (%)	57 (81.4)	59 (84.3)	0.411
Age, years, mean (SD)*	56.3 (12.1)	54.3 (17.5)	0.454
White race, n (%)	70 (100.0)	69 (98.6)	0.500
Educational level			0.985
Basic, n (%)	19 (27.1)	18 (26.5)	
Nonuniversity higher, n (%)	35 (50.0)	35 (50.0)	
University, n (%)	15 (22.1)	16 (22.9)	
Comorbidities			
Non-RA comorbidity, n (%)	64 (91.4)	50 (71.4)	0.002
No. of non-RA comorbidities, median (IQR)**	2.0 (2.0)	1.0 (2.0)	0.024
CCI, median (IQR)**	0.0 (1.0)	0.0 (0.0)	<0.001
ACCI, median (IQR)**	2.0 (2.0)	1.0 (1.5)	<0.001
Dyslipidemia, n (%)	16 (22.9)	10 (14.3)	0.139
Hypertension, n (%)	18 (25.7)	21 (30.0)	0.353
Smoking			0.037
Nonsmoker, n (%)	31 (44.3)	46 (65.7)	
Exsmoker, n (%)	18 (25.7)	12 (17.1)	
Active smoker, n (%)	21 (30.0)	12 (17.1)	
Diabetes mellitus, n (%)	8 (11.4)	6 (8.6)	0.390
Anxiety disorder, n (%)	15 (21.4)	4 (5.8)	0.007
Depression, n (%)	9 (12.9)	3 (4.3)	0.068
Clinical (only RA)			
Disease duration, median (IQR), months	75.9 (150.2)	NA	--
Diagnostic delay, median (IQR), months	7.0 (7.6)	NA	--
Erosions, n (%)	34 (48.6)	NA	--
Positive RF (>10 U/mL), n (%)	60 (85.7)	0 (0.0)	<0.001
Positive ACPA (>20 U/mL), n (%)	56 (80.0)	0 (0.0)	<0.001
ACPA >340 U/mL, n (%)	20 (28.6)	0 (0.0)	<0.001
DAS28-CRP at cut-off, mean (SD)	5.0 (1.1)	NA	--
Average cumulative DAS28-CRP, mean (SD)	3.7 (0.9)	NA	--
HAQ, median (IQR)	1.4 (0.9)	NA	--
Average HAQ, mean (SD)	1.1 (0.6)	NA	--
Anthropometric characteristics			
BMI, kg/m², mean (SD)*	27.4 (4.3)	27.3 (4.8)	0.952
Classification (WHO)			0.479
Normal weight (18.5-24.9), n (%)	20 (29.0)	23 (35.9)	
Overweight (25.0-29.9), n (%)	30 (43.5)	24 (37.5)	
Obesity grade I (30.0-34.5), n (%)	15 (21.7)	15 (23.4)	
Obesity grade II, n (%)	4 (5.8)	1 (1.6)	
Obesity grade III, n (%)	0 (0.0)	1 (1.6)	
Treatment (only RA)			
Methotrexate, n (%)	45 (64.3)	NA	--
Hydroxychloroquine, n (%)	11 (15.7)	NA	--
Leflunomide, n (%)	11 (15.7)	NA	--
Sulfasalazine, n (%)	19 (27.1)	NA	--
Corticosteroids, n (%)	52 (74.3)	NA	--
Corticosteroids, median (IQR)	5.0 (7.5)	NA	--

RA, rheumatoid arthritis; SD, standard deviation; IQR, interquartile range; CCI, Charlson comorbidity index; ACCI, age-adjusted Charlson comorbidity index; RF, rheumatoid factor; ACPA, anti–citrullinated peptide antibody; DAS28-CRP, 28-joint Disease Activity Score based on C-reactive protein; HAQ, Health Assessment Questionnaire; BMI, body mass index; WHO, World Health Organization; NA, not applicable.

Proportions were compared using the chi-squared test. Variables marked with an asterisk (*) were analyzed using the independent-samples t test; variables marked with 2 asterisks (**) were analyzed using the Mann-Whitney test.

Both groups were similar in terms of age, race, and educational level. However, comorbidities were more frequent in patients with RA than in controls (91.4% vs. 71.4%; p = 0.002). RA patients also had more comorbid conditions (p = 0.024) and higher scores in the CCI and ACCI (both p < 0.001), as well as a higher frequency of anxiety disorders (21.4% vs. 5.8%; p = 0.007). They also tended to have depression more frequently, although the difference was not significant (p = 0.068). The frequency of smoking differed between the groups (p = 0.037), with a greater prevalence of active smokers and exsmokers among patients with RA. No significant differences were found for anthropometric variables.

As for the specific clinical characteristics of RA, the median (IQR) disease duration was 75.9 (150.2) months, and the median diagnostic delay was 7.0 (7.6) months. Most patients had positive values for serum RF (85.7%) and ACPA (80%), with levels >340 U/mL in 28.6%. The mean DAS28-CRP index was 5.0 (1.1) at baseline, indicating moderate-high disease activity, and the median (IQR) HAQ score was 1.4 (0.9), indicating relevant functional involvement. The average cumulative values for both scales were lower, although they continued to indicate a moderate degree of activity and functional disability.

The markers of systemic inflammation confirmed a more intense systemic inflammatory state in RA, as follows: CRP (13.5 vs. 4.0 mg/L; p < 0.001), ESR (28.8 vs. 12.5; p < 0.001), and hemoglobin (slightly lower; p = 0.025).

As for concomitant treatment, 64.3% were taking methotrexate and 74.3% were taking corticosteroids (median 5.0 mg).

#### 3.1.2 Markers of inflammation, metabolic activity, and intestinal barrier integrity in patients and controls at baseline

**Table 2** compares the concentrations of the main markers of inflammation and intestinal barrier integrity and adipokines between patients and controls.

**Table 2:** Median (IQR) levels of biomarkers of inflammation and intestinal barrier integrity in serum and feces.

Marker	Patients (N = 70)	Controls (N = 70)	P value
Inflammatory markers			
CRP, mg/L, median (IQR)	13.5 (17.0)	4.0 (2.0)	<0.001
IL-6, pg/mL, median (IQR)	5.6 (9.2)	2.0 (1.4)	<0.001
IL-1β, median (IQR)	10.8 (9.3)	9.9 (8.9)	0.902
TNF- α, pg/mL, median (IQR)	31.1 (62.4)	32.5 (37.8)	0.464
Oxidized LDL, ng/mL, median (IQR)	114.6 (116.4)	86.4 (32.4)	<0.001
ESR, median (IQR)	26.0 (23.8)	11.0 (10.0)	<0.00
Hemoglobin, median (IQR)	13.1 (1.5)	13.3 (1.6)	0.076
Intestinal barrier integrity, serum			
Occludin, pg/mL, median (IQR)	36.0 (26.0)	42.6 (17.5)	<0.001
Claudin-1, pg/mL, median (IQR)	26.6 (35.4)	39.9 (29.3)	<0.001
Zonulin, ng/mL, median (IQR)	3.7 (5.2)	4.5 (4.7)	0.091
LPS, µg/mL, median (IQR)	106.9 (78.5)	167.4 (101.3)	<0.001
LBP, µg/mL, median (IQR)	3.2 (4.3)	4.5 (8.2)	0.011
Intestinal barrier integrity, feces			
Occludin, pg/mL, median (IQR)	51.2 (54.9)	43.5 (43.7)	0.608
Claudin-1, pg/mL, median (IQR)	57.7 (60.5)	64.1 (70.9)	0.406
Zonulin, ng/mL, median (IQR)	7.1 (6.8)	8.7 (9.5)	0.826
Adipokines			
Adiponectin, µg/mL, median (IQR)	12.8 (1.8)	11.5 (3.5)	0.001
Leptin, ng/mL, median (IQR)	16.6 (15.2)	18.3 (16.2)	0.293
Resistin, ng/mL, median (IQR)	6.1 (4.0)	5.6 (3.0)	0.056
Metabolism and growth			
IGF-1, µg/mL, mean (SD)	125.1 (19.5)	121.9 (15.1)	0.287

All p values correspond to the Mann-Whitney test for the comparison of medians between groups, except for IGF-1, which was compared using the independent-samples t test.

IQR, interquartile range; CRP, C-reactive protein; IL-6, interleukin 6; IL-1β, interleukin 1 beta; TNF-α, tumor necrosis factor alpha; LDL, low-density lipoprotein; ESR, erythrocyte sedimentation rate; LPS, lipopolysaccharide; LBP, LPS-binding protein; IGF-1, insulin-like growth factor type 1.

Compared with controls, patients with RA had significantly higher median (IQR) levels of CRP (13.5 [17.0] mg/L vs. 4.0 [2.0] mg/L; p < 0.001]), IL-6 (5.6 [9.2] pg/mL vs. 2.0 [1.4] pg/mL; p < 0.001), and oxidized LDL (114.6 [116.4] vs. 86.4 [32.4] ng/mL; p < 0.001). No significant differences were observed for baseline serum levels of IL-1β, TNF-α, or IGF-1.

As for serum markers of intestinal barrier integrity, patients with RA had significantly lower concentrations of occludin and claudin-1 (both p<0.001), whereas levels of zonulin were slightly higher, with no statistically significant differences (p=0.091). In contrast, levels of LPS and LBP were lower in patients with RA than in controls (p<0.001 and p=0.011, respectively).

No statistically significant differences between the groups were observed for concentrations of occludin, claudin, or zonulin in feces (all comparisons p > 0.05).

The analysis of adipokines revealed that adiponectin values were significantly higher in patients with RA (p = 0.001). No significant differences were observed for leptin (p = 0.293) or resistin (p = 0.056).

**Figure 1:**
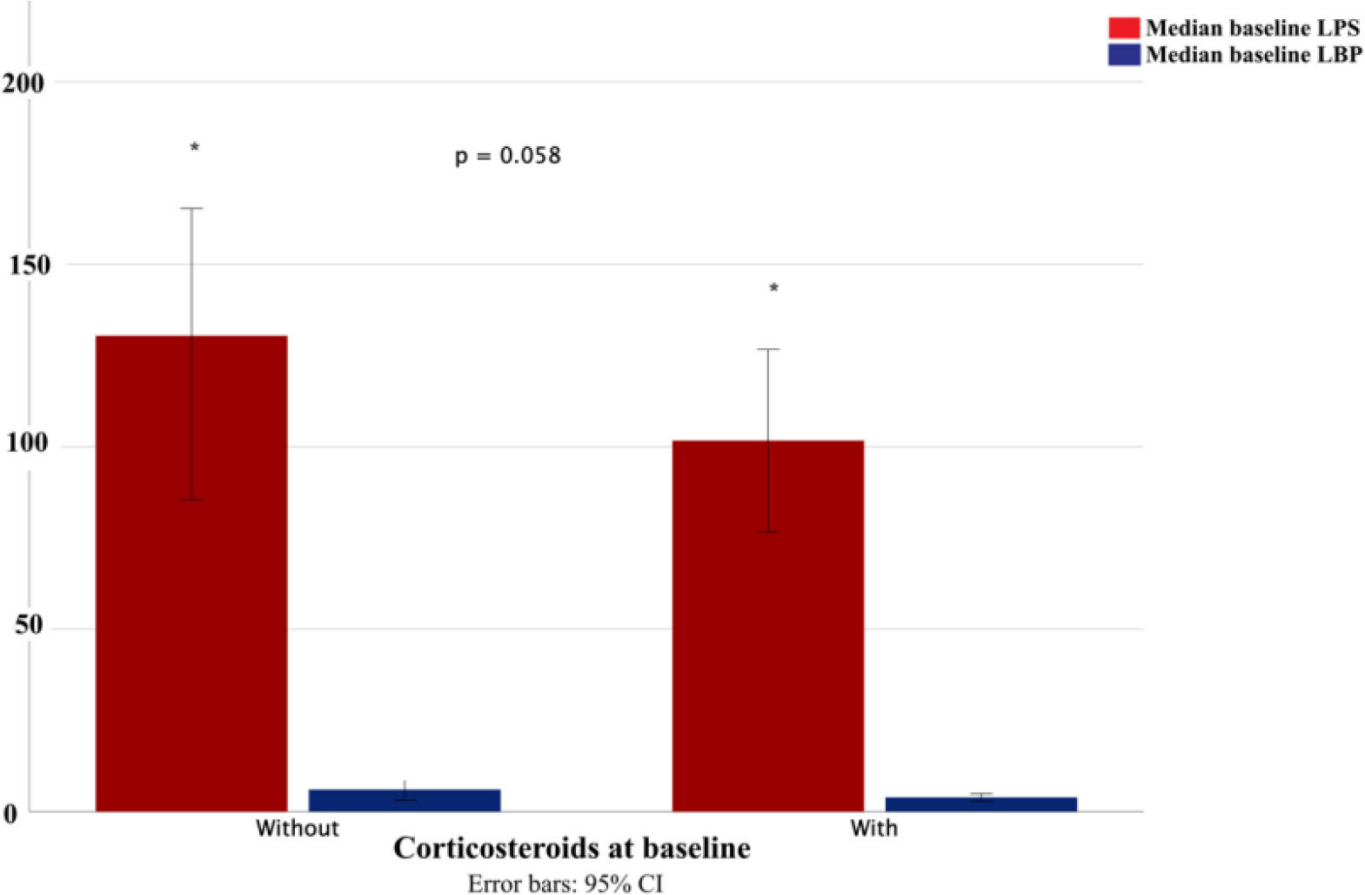
Levels of lipopolysaccharide (LPS) and LPS-binding protein (LBP) in serum according to corticosteroid therapy at baseline (Mann-Whitney test for LPS p = 0.058) in patients with rheumatoid arthritis. Statistical significance was considered when p < 0.05. * Indicates a trend toward significance (p = 0.058).

An additional exploratory analysis was conducted to evaluate the effect of corticosteroids on baseline levels of LPS and LBP in patients with RA. Those who received prednisone had numerically lower levels of LPS (median [IQR] 101.7 [69.7] vs. 149.1 [91.3] ng/mL; p = 0.058) and LBP (median [IQR] 2.7 [4.2] vs. 4.5 [6.6] ng/mL; p = 0.179) than those who did not, although the difference was not statistically significant (**Figure 1**).

### 3.2 Baseline correlations between biomarkers and clinical-laboratory variables

**Supplementary Table 1** shows the results for the baseline correlations between serum and fecal TJP levels and different clinical, inflammatory, and metabolic variables in patients with RA. Serum occludin levels correlated negatively with age (ρ = –0.286; p = 0.017), ACCI (ρ = –0.306; p = 0.011), RF levels (ρ = –0.272; p = 0.024), the HAQ score (ρ = –0.263; p = 0.030), IL-6 levels (ρ = –0.278; p = 0.020), and, more markedly, oxidized LDL activity (ρ = –0.591; p < 0.001).

A similar pattern was observed for claudin-1, that is, negative correlations with the CCI (ρ = –0.279; p = 0.020), RF levels (ρ = –0.340; p = 0.004), and oxidized LDL (ρ = –0.485; p < 0.001). In contrast with occludin, a positive association was observed between claudin-1 and TNF-α levels (ρ = 0.242; p = 0.043) and mean cumulative disease activity (average DAS28-CRP) (ρ = 0.325; p = 0.007).

Serum zonulin correlated positively with average DAS28-CRP (ρ = 0.375; p = 0.002) and negatively correlated with RF (ρ = –0.265; p = 0.028), resistin (ρ = –0.393; p = 0.001), and oxidized-LDL (ρ = –0.505; p < 0.001).

Fewer significant correlations were observed for biomarkers in feces. A negative correlation was observed between fecal claudin-1 and serum resistin (ρ = –0.277; p = 0.020), whereas positive correlations were observed between fecal zonulin and CRP (ρ = 0.327; p = 0.006) and IL-6 (ρ = 0.242; p = 0.006).

Lastly, a significant positive correlation was observed between serum and fecal levels of claudin-1 (ρ = 0.251; p = 0.036). No relevant correlations were observed between serum and fecal levels of zonulin.

### 3.3 Changes in symptoms and biomarkers after anti-TNF treatment

#### 3.3.1 Changes in symptoms, inflammation, and intestinal permeability after 6 months of treatment

**Table 3** shows the changes recorded in symptoms, inflammation, and intestinal permeability in patients with RA after 6 months of anti-TNF therapy, of whom 60 were treated with adalimumab (85.7%) and 10 with etanercept (14.3%). Symptoms improved significantly, with a reduction in the mean DAS28-CRP from 5.0 to 2.8 (p < 0.001). This improvement was consistent in all the individual components of the index, including the tender and swollen joint counts, and in the VAS scores for global activity, pain, and physician’s evaluation (all p < 0.001).

**Table 3:** Clinical, laboratory, and inflammatory variables and intestinal permeability in patients with RA: Results after 6 months of anti-TNF therapy.

Variable	Baseline 70 RA	6 months 70 RA	P value
Clinical			
DAS28-CRP response	–		
Remission, n (%)	NA	33 (47.1)	NA
Low activity, n (%)	NA	18 (25.7)	NA
Moderate activity, n (%)	NA	13 (18.6)	NA
High activity, n (%)	NA	6 (8.6)	NA
EULAR response			
Good response, n (%)	NA	41 (58.6)	NA
Moderate response, n (%)	NA	19 (27.1)	NA
No response, n (%)	NA	10 (14.3)	NA
DAS28-CRP, mean (SD)	5.0 (1.1)	2.8 (1.1)	<0.001
TJC, median (IQR)	6.0 (8.0)	1.0 (2.0)	<0.001
SJC, median (IQR)	3.0 (5.0)	0.0 (1.0)	<0.001
VAS general, median (IQR)	7.0 (3.0)	5.0 (4.0)	<0.001
VAS pain, median (IQR)	7.0 (2.8)	5.0 (4.0)	<0.001
VAS physician, median (IQR)	7.0 (2.0)	3.0 (3.0)	<0.001
HAQ, median (IQR)	1.4 (0.9)	1.0 (1.1)	<0.001
BMI, kg/m2, mean (SD)	27.4 (4.3)	27.3 (4.5)	<0.001
Prednisone, n (%)	52 (74.3%)	13 (18.6%)	<0.001
Prednisone, mg/d	7.5 (3.8)	5.0 (3.7)	0. 589
Markers of inflammation			
CRP, mg/L, median (IQR)	13.5 (17.0)	2.0 (9.0)	<0.001
IL-6, pg/mL, median (IQR)	5.6 (9.2)	2.8 (5.4)	0.006
IL-1β, median (IQR)	10.8 (9.3)	4.8 (3.4)	<0.001
TNF- α, pg/mL, median (IQR)	31.1 (62.4)	20.9 (7.0)	<0.001
Oxidized LDL, ng/mL, median (IQR)	114.6 (116.4)	86.3 (41.7)	<0.001
ESR, median (IQR)	26.0 (23.8)	19.0 (21.0)	0.022
Hemoglobin, median (IQR)	13.1 (1.5)	13.1 (1.5)	0.047
Intestinal barrier integrity, serum			
Occludin, pg/mL, median (IQR)	36.0 (26.0)	49.4 (57.4)	<0.001
Claudin-1, pg/mL, median (IQR)	26.6 (35.4)	35.8 (32.9)	<0.001
Zonulin, ng/mL, median (IQR)	3.7 (5.2)	3.9 (3.5)	0.179
LPS, µg/mL, median (IQR)	106.9 (78.5)	121.4 (178.7)	0.001
LBP, µg/mL, median (IQR)	3.2 (4.3)	10.3 (2.5)	<0.001
Intestinal barrier integrity, feces			
Occludin, pg/mL, median (IQR)	51.2 (54.9)	45.3 (47.4)	0.122
Claudin-1, pg/mL, median (IQR)	57.7 (60.5)	78.4 (36.4)	0.130
Zonulin, ng/mL, median (IQR)	7.1 (6.8)	11.8 (15.6)	<0.001
Adipokines			
Adiponectin, µg/mL, median (IQR)	12.8 (1.8)	16.4 (2.8)	<0.001
Leptin, ng/mL, median (IQR)	16.7 (15.2)	17.3 (25.9)	0.001
Resistin, ng/mL, median (IQR)	6.1 (4.0)	8.2 (5.2)	<0.001
Metabolism and growth			
IGF-1, µg/mL, mean (SD)	125.1 (19.5)	152.4 (26.8)	<0.001

RA, rheumatoid arthritis; TNF-α, tumor necrosis factor alpha; DAS28-CRP, 28-joint Disease Activity Score based on C-reactive protein; EULAR, European League Against Rheumatism; SD, standard deviation; TJC, tender joint count; IQR, interquartile range; SJC, swollen joint count; VAS, visual analog scale; HAQ, Health Assessment Questionnaire; BMI, body mass index; CRP, C-reactive protein; IL-6, interleukin 6; IL-1β, interleukin 1 beta; LDL, low-density lipoprotein; ESR, erythrocyte sedimentation rate; LPS, lipopolysaccharide; LBP, LPS-binding protein; IGF-1, insulin-like growth factor 1.

All p values for the quantitative variables correspond to the Wilcoxon test for the comparison of baseline and 6-month median values, except for DAS28-CRP, BMI, and IGF-1, which were compared using the paired-samples t test. The percentage of patients taking prednisone was analyzed using the McNemar test.

Clinical remission was achieved by 47.1% of patients (DAS28-CRP <2.6), and a good response according to the EULAR criteria was achieved by 58.6%. Physical function also improved significantly, with a decrease in the median HAQ score from 1.4 to 1.0 (p < 0.001), indicating reduced functional disability.

As for concomitant treatment, a marked reduction was observed in the use of prednisone, falling from 74.3% to 18.6% of patients (p < 0.001, McNemar test). The median dose fell from 7.5 to 5.0 mg/d, although the difference was not statistically significant (p = 0.589, Wilcoxon test).

In terms of inflammation, a significant decrease was observed for levels of IL-6 (p = 0.006), IL-1β, TNF-α, CRP, oxidized-LDL, and ESR (all p < 0.05), pointing to a more favorable inflammatory profile after treatment. In parallel, a significant increase was observed for adiponectin, leptin, resistin, and IGF-1 (all p < 0.001), indicating potential metabolic effects for anti-TNF therapy.

As for biomarkers of intestinal permeability, a significant increase was observed for serum occludin and claudin-1 (both p<0.001), as well as for serum LPS (p = 0.001) and LBP (p < 0.001). No significant changes were found in serum zonulin levels (p = 0.179). In feces, zonulin levels increased significantly (p < 0.001), whereas no statistically significant differences were observed for those of occludin and claudin-1.

#### 3.3.2 Baseline characteristics associated with clinical remission after anti-TNF therapy

At 6 months of treatment, 47% of RA patients achieved clinical remission. As observed in **Supplementary Table 2**, no significant differences at baseline were recorded between the groups (remission vs no remission) for sociodemographic variables, general comorbidity, or concomitant treatment. However, anxiety disorders were less common in the remission group (9.1% vs. 32.4%, p = 0.017), and affected patients had less functional disability at initiation of treatment, with lower baseline HAQ scores (median 1.0 vs. 1.7; p = 0.006) and average HAQ scores (p = 0.014).

As for biomarkers, no significant differences were found for inflammatory parameters, adipokines, or metabolic profile. Nevertheless, patients who achieved remission had significantly higher levels of occludin in serum and feces (p = 0.033 and p = 0.030, respectively) and claudin-1 in feces (p = 0.012), suggesting better intestinal barrier integrity in this group.

### 3.4 Multivariate analysis

**Table 4** shows the results of the multivariate logistic regression analysis performed to identify the baseline factors associated with anti-TNF therapy in patients with RA. The analysis showed that a higher HAQ scores throughout follow-up was significantly associated with a lower probability of achieving clinical remission (OR = 0.26; 95% CI: 0.09–0.74; p = 0.011). In contrast, higher baseline levels of serum occludin were associated with a greater probability of remission (OR = 1.04; 95%CI: 1.01–1.08; p = 0.025) as well as with higher baseline levels of claudin-1 in feces (OR 1.02: 95%CI: 1.00-1.02; P = 0.017). The model explained 35.1% of the variability of the dependent variable (Nagelkerke R² = 0.351). The variables included in the equation were age, sex, disease duration, smoking, average DAS28-CRP, average HAQ, serum occludin and fecal claudin-1.

**Table 4:** Multivariate analysis of baseline characteristics associated with remission in RA after 6 months of anti-TNF therapy.

Predictor	Univariate			Multivariate		
	OR	95% CI	P value	OR	95% CI	P value
Age, years	0.98	0.95–1.02	0.396	—	—	—
Female sex	1.01	0.30–3.40	0.986	—	—	—
History of smoking, active smokers, and exsmokers	2.18	0.66–7.12	0.223	—	—	—
Average DAS28-CRP	0.70	0.41–1.17	0.172	—	—	—
Average HAQ	0.32	0.13–0.77	0.012	0.26	0.09-0.74	0.011
Baseline serum occludin, pg/mL	1.04	1.01–1.08	0.018	1.04	1.01-1.08	0.025
Baseline feces claudin-1, pg/mL	1.01	1.00-1.02	0.020	1.02	1.00-1.02	0.017

Nagelkerke R^2^ = 0.351. The variables included in the equation were sex, age, history of smoking, average DAS28-CRP, average HAQ, serum occludin and claudin-1 in feces.

RA, rheumatoid arthritis; TNF, tumor necrosis factor alpha; OR, odds ratio; CI, confidence interval; DAS28-CRP, 28-joint Disease Activity Score based on C-reactive protein; HAQ, Health Assessment Questionnaire.

Three multivariate linear regression models were then constructed to explore the baseline factors associated with serum biomarkers of intestinal barrier integrity after 6 months of treatment (Δ Occludin, Δ Claudin-1, and Δ Zonulin).

In the first model (**Table 5**), the increase in serum occludin levels after anti-TNF therapy was significantly associated with greater baseline inflammatory activity (DAS28-CRP: β = 0.314; p = 0.003) and higher levels of IL-1β at initiation of therapy (β = 0.416; p < 0.001). This model explained 27.3% of the variability observed (adjusted R² = 0.273).

**Table 5:** Multivariate analysis of changes in serum occludin in patients with RA 6 months after treatment with anti-TNF agents.

Predictor	Univariate			Multivariate		
	B	95% CI	P value	B	95% CI	P value
Age, years	0.21	-0.49, 0.91	0.550	—	—	—
Female sex	14.89	-6.68, 36.45	0.173	—	—	—
History of smoking, active smokers, and exsmokers	-8.74	-18.65, 1.46	0.092	—	—	—
Baseline prednisone	13.86	-5.31, 33.02	0.154	—	—	—
Baseline DAS28-CRP	11.35	3.99, 18.70	0.003	10.19	3.52, 16.86	0.003
TNF-α pg/mL	0.03	-0.01, 0.06	0.209	—	—	—
Baseline IL-1β, pg/mL	2.29	1.17, 3.41	<0.001	2.15	1.09, 3.21	<0.001

Adjusted R^2^ = 0.273. The variables included in the analysis were sex, age, history of smoking, baseline prednisone, baseline DAS28-CRP, and baseline levels of TNF-α and IL-1β.

RA, rheumatoid arthritis; TNF, tumor necrosis factor alpha; CI, confidence interval; DAS28-CRP, 28-joint Disease Activity Score based on C-reactive protein.

In the second model (**Table 6**), the only factor that was significantly associated with the change in serum claudin-1 level after anti-TNF therapy was sex: levels increased by more after treatment in men (β = –0.342; p = 0.004). The model accounted for 11.7% of the variability (adjusted R² = 0.117).

**Table 6:** Multivariate analysis of changes in serum claudin-1 in RA after 6 months of treatment with anti-TNF agents.

Predictor	Univariate	95% CI	P value	Multivariate	95% CI	P value
	B			B		
Age, years	0.04	(-0.65, 0.74)	0.896	—	—	—
Female sex	-30.57	(-50.89, -10.24)	0.004	-30.57	(-50.89, -10.24)	0.004
History of smoking, active smokers, and exsmoker	-1.18	(-11.48, 0.13)	0.821	—	—	—
Baseline prednisone	-0.26	(-19.50, 18.99)	0.979	—	—	—
Baseline DAS28-CRP	0.52	(-7.24, 8.29)	0.893	—	—	—
Positive RF	20.43	(-3.09, 43.95)	0.088	—	—	—
Oxidized LDL, IU/mL	0.01	(-0.08, 0.09)	0.914	—	—	—

Adjusted R^2^ = 0.117. The variables included in the equation were age, sex, smoking, baseline prednisone, baseline DAS28-CRP, positive RF, and baseline oxidized LDL levels.

RA, rheumatoid arthritis; TNF, tumor necrosis factor alpha; CI, confidence interval; DAS28-CRP, 28-joint Disease Activity Score based on C-reactive protein; RF, rheumatoid factor; LDL, low-density lipoprotein.

In the third model (**Table 7**), the increase in serum zonulin levels after anti-TNF therapy was associated with lower IL-1β values (β = –0.313; p = 0.007) and higher resistin levels at initiation of treatment (β = 0.294; p = 0.010). This model accounted for 18.5% of the variability (adjusted R² = 0.185).

**Table 7:** Multivariate analysis of changes in serum zonulin in patients with RA after 6 months of anti-TNF therapy.

Predictor	Univariate	95% CI	P-value	Multivariate	95% CI	P value
	B			B		
Age, years	0.06	(-0.02, 0.14)	0.166	—	—	—
Female sex	1.5	(-1.06, 4.06)	0.246	—	—	—
History of smoking, active smokers, and exsmokers	0.57	(-0.66, 1.79)	0.357	—	—	—
Baseline prednisone	0.32	(-1.98, 2.62)	0.784	—	—	—
Average DAS28-CRP	-1.3	(-2.34, -0.27)	0.015	—	—	—
IL-1β	-0.22	(-0.36, -0.09)	0.002	-0.19	(-0.32, -0.06)	0.007
Resistin, ng/mL	0.55	(0.17, 0.92)	0.005	-0.47	(0.11, 0.83)	0.01

Adjusted R^2^ = 0.185. The variables included in the equation were age, sex, smoking history, baseline prednisone, average DAS28, IL-1β level, and baseline resistin levels.

RA, rheumatoid arthritis; TNF, tumor necrosis factor alpha; CI, confidence interval; DAS28-CRP, 28-joint Disease Activity Score based on C-reactive protein; IL-1β, interleukin 1 beta.

## 4 Discussion

Zonulin and TJPs regulate intestinal permeability and could be potential biomarkers of intestinal barrier integrity and permeability (23). Although a few studies have addressed this issue in RA patients treated with DMARDs, to our knowledge this study is the first to study the issue prospectively and in a controlled manner.

This study shows that patients with RA exhibit disrupted intestinal barrier integrity, evidenced by lower serum occludin and claudin−1 compared with HC, and that improvement in systemic inflammation under TNF inhibition is accompanied by normalization of these biomarkers among patients who achieve remission. Higher baseline serum levels of occludin and fecal claudin-1 were associated with a greater likelihood of remission, suggesting that a relatively preserved tight epithelial seal confers greater reversibility of barrier dysfunction under anti-TNF therapy.

Normalization of occludin and claudin-1 values after 6 months of anti-TNF therapy, together with improved symptoms and reduced inflammatory parameters, reinforces the potential role of these biomarkers, not only as indicators of damage, but also as potential indicators of treatment response. These biomarkers are not merely bystanders, but they are mechanistically linked to RA disease activity.

These data are consistent with the limited bibliographic data on TJPs in inflammatory rheumatic diseases in general and highlight the importance of intestinal barrier dysfunction in the amplification, and even the triggering, of the autoimmune response. From a mechanistic perspective, disruption of tight junction organization may facilitate increased exposure to luminal antigens and microbe-associated molecular patterns, which can activate innate immune pathways such as Toll-like receptor signaling and downstream production of pro-inflammatory cytokines including TNF, IL-6 and IL-1. These mediators are central to RA pathogenesis and can promote adaptive immune responses such as Th17 differentiation and autoantibody production, providing a plausible link between intestinal barrier alterations and systemic autoimmunity. Conversely, systemic inflammation and TNF-driven pathways can themselves modulate tight junction protein expression and localization, suggesting a bidirectional interaction in which barrier dysfunction and immune activation reinforce each other (24–27).

Recent studies have identified similar reductions in occludin and claudin in patients with ulcerative colitis and Crohn disease, conditions in which the gut–systemic immunity axis is already well established (28). While evidence in RA continues to develop, the findings reported here highlight the importance of investigating therapeutic modulators that restore TJP function as a strategy for improving clinical response and long-term prognosis.

Zonulin showed inconsistent behavior, related to the analytical limitations of current assays and variable correlation with direct permeability tests; interpretation should therefore remain cautious. These apparent inconsistences have also been reported in inflammatory diseases other than RA, such as inflammatory bowel disease, and could be interpreted as an adaptive or compensatory effect against treatment-induced immune modulation, as well as against other factors that limit the specificity and sensitivity of this biomarker of intestinal barrier integrity. In this sense, it must be stressed that commercial zonulin ELISA kits also detect other members of the family, namely, zonulin-related proteins, and underestimate the presence of zonulin and its analogues (26, 27, 29). Moreover, previous studies have shown that serum zonulin levels are inconsistently correlated with direct intestinal permeability tests, thus limiting their application in clinical practice (30–32).

In contrast to expectations for a chronic inflammatory condition, patients with RA showed lower baseline serum levels of LPS and LBP than healthy controls. Several factors may explain this finding. Total circulating LPS reflects both bioactive and inactive fractions, and we did not assess LPS bioactivity, endotoxin-neutralizing antibodies, or downstream signaling activity. Importantly, total LPS does not necessarily represent its true proinflammatory potential, as a substantial proportion is bound to lipoproteins or neutralizing proteins. These biological and technical constraints make LPS and LBP controversial biomarkers (33). Furthermore, circulating LPS is rapidly redistributed and neutralized by LBP, soluble CD14, and high-density lipoproteins, which affects both its biological activity and its detectability in serum (34, 35).

Assessment of serum Toll-like receptor 4 (TLR4)–stimulating activity has been proposed as a more functionally relevant alternative, as it better reflects endotoxin-induced immune activation and has been associated with inflammatory parameters and disease activity in RA (12).

In addition, the widespread use of systemic glucocorticoids as bridging therapy prior to anti-TNF initiation may have influenced circulating LPS and LBP levels, given their modulatory effects on endotoxin-related pathways (36). In our exploratory analyses, patients receiving prednisone tended to show lower LPS and LBP concentrations than those not treated with corticosteroids, although these differences were not statistically significant.

Taken together, these considerations limit the interpretation of LPS/LBP measurements as direct indicators of bacterial translocation in RA. Therefore, our findings should be regarded as exploratory and hypothesis-generating rather than conclusive evidence of altered endotoxin burden.

Finally, it included several secondary multivariate analyses that reinforce the importance of intestinal barrier integrity and inflammation as determinants of remission and prognosis in RA, and the interrelationships between inflammation, epithelial barrier integrity, and anti-TNF treatment, and suggest that tight junction biomarkers may be useful in patient stratification and follow-up (26, 28).

The interpretation of the present findings is subject to several limitations. First, the sample size may have limited the statistical power to detect certain associations and to precisely estimate relationships between intestinal integrity biomarkers and clinical-inflammatory parameters. However, the prospective and controlled design allowed the detection of between-group differences, treatment-related changes, and the identification of potential response biomarkers.

Second, the absence of direct functional permeability tests (e.g., lactulose-mannitol assay or assessment of LPS bioactivity) limits the physiological validation of these surrogate biomarkers. Therefore, occludin, claudin-1, zonulin, and LPS/LBP should be interpreted as indirect indicators of epithelial barrier status rather than direct measurements of gut permeability or bacterial translocation.

Residual confounding factors that were not measured (e.g., genetic background, dietary patterns, stress, or previous medication exposure) cannot be completely excluded. Nevertheless, the potential effect of bridging glucocorticoid therapy was explored, and the strict inclusion criteria (biologic-naïve patients, stable diet, no recent antibiotics or probiotics) contributed to improving sample homogeneity.

External validation of the sample is lacking, and occludin and claudin-1 levels require confirmation in larger, independent cohorts. Finally, the observational design precludes causal inference regarding the bidirectional relationship between barrier dysfunction and systemic inflammation.

In conclusion, compared with healthy controls, patients with RA showed alterations in biomarkers related to intestinal barrier integrity, including structural markers such as occludin and claudin-1, as well as the regulatory protein zonulin. These alterations are associated with a more active inflammatory state and seem to be reversible, at least partially, after 24 weeks of anti-TNF therapy. Furthermore, the baseline differences in specific biomarkers between patients who achieved clinical remission and those who did not suggest a possible predictive value for certain components of the intestinal barrier in the response to therapy. These findings support the hypothesis that intestinal barrier dysfunction could play a relevant role in the pathogenesis of RA and that restoration of barrier integrity could help to modulate systemic inflammation. In addition, they pave the way for new lines of research into the role of the intestinal barrier in the pathogenesis of RA and the response to therapy. Longitudinal studies with larger samples and functional measurements of intestinal permeability would make it possible to confirm the prognostic value of biomarkers such as serum occludin and to explore therapeutic strategies aimed at restoring barrier integrity as a complement to conventional immunomodulatory treatment. These would in turn contribute to the provision of more personalized medicine for treatment of RA. 

## Data Availability

The raw data supporting the conclusions of this article will be made available by the authors, without undue reservation.
