# Supplementary material for: Association between intestinal permeability, systemic inflammation, and response to anti-TNF therapy in patients with rheumatoid arthritis: a prospective controlled study
Source: Front Immunol. 2026 Mar 27;17:1756621. doi: 10.3389/fimmu.2026.1756621 (PMC13067904; doi:10.3389/fimmu.2026.1756621)
Supplement: Supplementary file 1 [file Table1.docx]

**Supplementary Material**

Supplementary table 1: Correlations between baseline intestinal permeability and clinical, laboratory, and inflammatory variables.

| Variable | Occludin in serum | Claudin-1 in serum | Zonulin in serum | Occludin in feces | Claudin-1 in feces | Zonulin in feces |
| --- | --- | --- | --- | --- | --- | --- |
| Age, years | -0.286* | -0.195 | -0.171 | -0.138 | -0.002 | 0.069 |
| Disease duration, months | -0.139 | -0.070 | -0.089 | -0.081 | 0.070 | 0.148 |
| Age-adjusted Charlson comorbidity index | -0.306* | -0.279* | -0.169 | -0.150 | -0.089 | -0.060 |
| Baseline BMI, kg/m^2^ | -0.007 | 0.045 | 0.096 | 0.117 | 0.084 | -0.023 |
| Corticosteroids at baseline, mg/d | -0.142 | -0.100 | 0.005 | -0.004 | 0.199 | 0.081 |
| ACPA, U/mL | 0.161 | 0.088 | 0.160 | 0.231 | -0.061 | 0.020 |
| RF, U/mL | -0.272* | -0.340** | -0.265** | -0.020 | 0.050 | 0.031 |
| DAS28-CRP | -0.099 | -0.057 | -0.033 | 0.121 | 0.126 | 0.019 |
| Average DAS28-CRP | 0.238 | 0.325** | 0.375** | 0.050 | 0.167 | 0.041 |
| HAQ | -0.263* | -0.190 | -0.206 | 0.061 | 0.029 | 0.028 |
| Average HAQ | -0.084 | -0.023 | -0.018 | -0.052 | 0.007 | -0.002 |
| Leptin, ng/mL | 0.038 | 0.051 | -0.107 | 0.010 | 0.063 | 0.079 |
| Resistin, ng/mL | -0.138 | -0.108 | -0.393** | 0.062 | -0.277* | 0.151 |
| Adiponectin, µg/mL | -0.124 | -0.173 | -0.110 | 0.159 | 0.158 | 0.022 |
| IL-6, pg/mL | -0.278* | -0.094 | -0.229 | 0.045 | -0.124 | 0.242* |
| CRP, mg/L | -0.187 | -0.112 | -0.161 | 0.142 | 0.024 | 0.327** |
| ESR, mm/h | -0.011 | 0.006 | -0.052 | 0.007 | 0.074 | 0.090 |
| IL-1β, pg/mL | 0.153 | 0.168 | 0.209 | 0.062 | -0.166 | 0.090 |
| IGF-1, pg/mL | 0.010 | 0.109 | -0.025 | 0.098 | 0.118 | 0.025 |
| Oxidized LDL (IU/mL) | -0.591** | -0.485** | -0.505** | -0.120 | -0.105 | 0.002 |
| TNF-α, pg/mL | 0.203 | 0.242* | 0.188 | 0.056 | -0.057 | 0.040 |

*Note: The Spearman correlation coefficient was used to evaluate the correlation between tight junction proteins and the different variables. * p < 0.05; ** p < 0.01.*

*Abbreviations: BMI: body mass index ACPA: anti–citrullinated antipeptide antibody;; RF: rheumatoid factor; DAS28-CRP: 28-joint Disease Activity Score based on C-reactive protein; HAQ: Health Assessment Questionnaire; IL-6: interleukin 6; CRP: C-reactive protein; ESR: erythrocyte sedimentation rate; IL-1β: interleukin 1 beta; IGF-1: insulin-like growth factor 1; LDL: low-density lipoprotein; TNF-α: tumor necrosis factor alpha.*

Supplementary table 2: Clinical characteristics and intestinal permeability in 70 patients with RA according to clinical remission status after 6 months of anti-TNF therapy.

| Variable | Remission N=33 | No remission N=37 | p value |
| --- | --- | --- | --- |
| Epidemiological |  |  |  |
| Female sex, n (%) | 30 (81.1) | 27 (81.8) | 0.591 |
| Age, years, mean (SD) | 55.0 (13.7) | 57.4 (10.8) | 0.434 |
| White race, n (%) | 33 (100.0) | 33 (100.0) | 1 |
| Educational level: |  |  | 0.689 |
| Basic, n (%) | 8 (24.5) | 11 (29.7) |  |
| Nonuniversity higher, n (%) | 16 (48.5) | 19 (51.4) |  |
| University, n (%) | 9 (27.3) | 7 (18.9) |  |
| Comorbidities |  |  |  |
| No. of non-RA comorbidities, median (IQR) | 2.0 (2.0) | 2.0 (3.0) | 0.644 |
| Charlson comorbidity index, median (IQR) | 1.0 (1.0) | 1.0 (1.0) | 0.523 |
| Age-adjusted Charlson comorbidity index, median (IQR) | 2.5 (3.0) | 2.0 (1.0) | 0.980 |
| Dyslipidemia, n (%) | 7 (21.2) | 9 (24.3) | 0.492 |
| Hypertension, n (%) | 8 (24.2) | 10 (27.0) | 0.504 |
| Diabetes mellitus, n (%) | 6 (18.2) | 2 (5.4) | 0.096 |
| Smoking |  |  | 0.264 |
| Nonsmoker, n (%) | 18 (54.5) | 13 (35.1) |  |
| Exsmoker, n (%) | 7 (21.2) | 11 (29.7) |  |
| Active smoker, n (%) | 8 (24.2) | 13 (35.1) |  |
| Anxiety disorder, n (%) | 3 (9.1) | 12 (32.4) | 0.017 |
| Depression, n (%) | 6 (16.2) | 3 (9.1) | 0.300 |
| BMI, kg/m2, mean (SD) | 27.5 (4.2) | 27.2 (4.4) | 0.764 |
| Clinical |  |  |  |
| Disease duration, median (IQR), months | 93.8 (127.4) | 60.3 (173.9) | 0.348 |
| Diagnostic delay, median (IQR), months | 5.8 (6.8) | 8.0 (9.4) | 0.144 |
| Erosions, n (%) | 19 (57.6) | 15 (40.5) | 0.118 |
| Positive RF (>10 U/mL), n (%) | 27 (81.8) | 33 (89.2) | 0.295 |
| Positive ACPA (>20 U/mL), n (%) | 29 (87.9) | 27 (73.0) | 0.104 |
| High ACPA >340 U/mL, n (%) | 10 (30.3) | 10 (27.0) | 0.484 |
| DAS28-CRP, mean (SD) | 4.9 (1.1) | 5.0 (1.1) | 0.753 |
| Average DAS28-CRP, median (IQR) | 3.4 (1.5) | 3.6 (1.2) | 0.236 |
| HAQ, median (IQR) | 1.0 (0.8) | 1.7 (1.0) | 0.006 |
| Average HAQ, mean (SD) | 0.9 (0.6) | 1.2 (0.6) | 0.014 |
| Treatment |  |  |  |
| Methotrexate, n (%) | 20 (60.6) | 25 (67.6) | 0.360 |
| Hydroxychloroquine, n (%) | 4 (12.1) | 7 (18.9) | 0.328 |
| Leflunomide, n (%) | 3 (9.1) | 8 (21.6) | 0.133 |
| Sulfasalazine, n (%) | 11 (33.3) | 8 (21.6) | 0.203 |
| Corticosteroids, median (IQR) | 5 (5.0) | 5 (6.3) | 0.128 |
| Corticosteroids, n (%) | 24 (72.7) | 28 (75.7) | 0.496 |
| Inflammatory markers |  |  |  |
| CRP, mg/L, median (IQR) | 13.0 (16.0) | 14.0 (18.5) | 0.972 |
| IL-6, pg/mL, median (IQR) | 6.0 (8.3) | 4.6 (12.0) | 0.499 |
| IL-1β, median (IQR) | 10.9 (8.8) | 9.0 (9.3) | 0.906 |
| TNF- α, pg/mL, median (IQR) | 34.1 (62.2) | 31.0 (66.9) | 0.473 |
| Oxidized LDL, ng/mL, median (IQR) | 113.5 (143.7) | 116.4 (110.7) | 0.410 |
| ESR, median (IQR) | 24.0 (23.5) | 27.0 (26.0) | 0.985 |
| Hemoglobin, median (IQR) | 13.0 (1.4) | 13.1 (1.6) | 0.925 |
| Intestinal barrier integrity, serum |  |  |  |
| Occludin, pg/mL, median (IQR) | 40.8 (19.1) | 30.9 (29.9) | 0.033 |
| Claudin-1, pg/mL, median (IQR) | 35.6 (30.3) | 18.8 (35.5) | 0.190 |
| Zonulin, ng/mL, median (IQR) | 4.7 (6.0) | 3.2 (5.0) | 0.131 |
| LPS, µg/mL, median (IQR) | 95.1 (75.4) | 126.7 (75.4) | 0.110 |
| LBP, µg/mL, median (IQR) | 3.5 (3.6) | 2.8 (6.0) | 0.537 |
| Intestinal barrier integrity, feces |  |  |  |
| Occludin, pg/mL, median (IQR) | 74.3 (59.2) | 38.1 (41.0) | 0.030 |
| Claudin-1, pg/mL, median (IQR) | 87.0 (68.5) | 46.8 (36.4) | 0.012 |
| Zonulin, ng/mL, median (IQR) | 6.9 (6.6) | 7.2 (8.1) | 0.869 |
| *Adipokines* |  |  |  |
| Adiponectin, µg/mL, median (IQR) | 12.8 (2.2) | 12.6 (2.2) | 0.948 |
| Leptin, ng/mL, median (IQR) | 18.2 (13.1) | 14.3 (17.2) | 0.420 |
| Resistin, ng/mL, median (IQR) | 5.7 (3.8) | 6.9 (4.8) | 0.462 |
| Metabolism and growth |  |  |  |
| IGF-1, µg/mL, mean (SD) | 124.9 (18.1) | 125.2 (20.8) | 0.950 |

*Abbreviations:* *RA: rheumatoid arthritis; TNF-α: tumor necrosis factor alpha; SD: standard deviation; IQR: interquartile range; BMI: body mass index; RF: rheumatoid factor; ACPA: anti–citrullinated peptide antibody; DAS28-CRP: 28-joint Disease Activity Score based on C-reactive protein; HAQ: Health Assessment Questionnaire; CRP: C-reactive protein; IL-6: interleukin 6; IL-1β: interleukin 1 beta; LDL: low-density lipoprotein; ESR: erythrocyte sedimentation rate; LPS: lipopolysaccharide; LBP: LPS-binding protein; IGF-1: insulin-like growth factor 1.*

*Complementary analysis: Clinical and laboratory differences by sex.*

While no differences were observed in the percentage of women among patients who achieved and did not achieve clinical remission (47.4% vs. 46.2%; p = 0.591), the analysis by sex revealed several differences in clinical and laboratory data (see Supplementary table 3). At initiation of the study, women had significantly higher levels of IGF-1 (median: 128.0 vs. 108.0; p = 0.001) and lower hemoglobin concentrations (12.7 [1.6] vs. 13.9 [1.3]; p < 0.001) than men.

Hydroxychloroquine was also prescribed more frequently to women (19.3% vs. 0%), although the difference was not statistically significant (p = 0.085). In contrast, active or previous smoking was more common in men (84.7% vs. 49.1%; p = 0.050). Moreover, women had a significantly lower ACCI (1.0 [0.75] vs. 2.0 [2.0]; p = 0.017).

During follow-up, female sex was associated with greater mean inflammatory activity, seen as a significantly higher average DAS28-CRP (3.8 [1.3] vs. 3.4 [1.0]; p = 0.018).

Supplementary table 3: Clinical characteristics and intestinal permeability in 70 patients with RA and response to anti-TNF therapy at 6 months according to sex.

| Variable | Women N=57 | Men N=13 | p value |
| --- | --- | --- | --- |
| Epidemiological |  |  |  |
| Age, years, mean (SD) | 55.7 (12.8) | 58.4 (9.8) | 0.482 |
| Educational level: |  |  | 0.551 |
| Basic, n (%) | 14 /24.6) | 5 (38.5) |  |
| Higher, nonuniversity, n (%) | 29 (50.9) | 6 (46.2) |  |
| University, n (%) | 14 (24.6) | 2 (15.4) |  |
| Comorbidities |  |  |  |
| No. of non-RA comorbidities, median (IQR) | 2.0 (3.0) | 2.0 (3.0) | 0.493 |
| Charlson comorbidity index, median (IQR) | 1.0 (0.75) | 2.0 (2.0) | 0.017 |
| Age-adjusted Charlson comorbidity index, median (IQR) | 2.0 (2.0) | 2.0 (3.0) | 0.204 |
| Dyslipidemia, n (%) | 14 (24.6) | 2 (15.4) | 0.718 |
| Hypertension, n (%) | 16 (28.1) | 2 (15.4) | 0.491 |
| Diabetes mellitus, n (%) | 5 (8.8) | 3 (23.1) | 0.161 |
| Smoking |  |  | 0.050 |
| Nonsmoker, n (%) | 29 (50.9) | 2 (15.4) |  |
| Exsmoker, n (%) | 12 (21.1) | 6 (46.2) |  |
| Smoker, n (%) | 16 (28.1) | 5 (38.5) |  |
| Anxiety disorder, n (%) | 13 (22.8) | 2 (15.4) | 0.720 |
| Depression, n (%) | 7 (12.3) | 2 (15.4) | 0.670 |
| BMI, kg/m2, mean (SD) | 27.4 (4.1) | 27.4 (5.2) | 0.981 |
| Clinical |  |  |  |
| Disease duration, median (IQR), meses | 69.2 (151.6) | 111.0 (169.0) | 0.902 |
| Diagnostic delay, median (IQR) meses | 7.0 (9.2) | 6.8 (7.0) | 0.297 |
| Erosions, n (%) | 26 (45.6) | 8 (61.5) | 0.233 |
| Positive RF (>10 U/mL), n (%) | 48 (84.2) | 12 (92.3) | 0.404 |
| Positive ACPA (>20 U/mL), n (%) | 45 (78.9) | 11 (84.6) | 0.490 |
| High ACPA (>340 U/mL), n (%) | 16 (28.1) | 4 (30.8) | 0.545 |
| DAS28-CRP, mean (SD) | 5.0 (1.1) | 4.8 (0.9) | 0.433 |
| Average cumulative DAS28-CRP, mean (SD) | 3.8 (1.0) | 3.3 (0.6) | 0.018 |
| HAQ, median (IQR) | 1.5 (0.9) | 1.0 (1.0) | 0.421 |
| Average HAQ, mean (SD) | 1.1 (0.6) | 0.9 (0.6) | 0.219 |
| Treatment |  |  |  |
| Methotrexate, n (%) | 38 (66.7) | 7 (53.8) | 0.523 |
| Hydroxychloroquine, n (%) | 11 (19.3) | 0 (0.0) | 0.085 |
| Leflunomide, n (%) | 9 (15.8) | 2 (15.4) | 0.669 |
| Sulfasalazine, n (%) | 15 (26.3) | 4 (30.8) | 0.494 |
| Corticosteroids, median (IQR) | 5.0 (2.5) | 5.0 (5.0) | 0.775 |
| Corticosteroids, n (%) | 43 (75.4) | 9 (69.2) | 0.442 |
| Inflammatory markers |  |  |  |
| CRP, mg/L, median (IQR) | 13.0 (15.8) | 20.0 (33.8) | 0.308 |
| IL-6, pg/mL, median (IQR) | 5.8 (14.1) | 5.3 (8.4) | 0.410 |
| IL-1β, median (IQR) | 10.4 (9.1) | 12.9 (10.1) | 0.689 |
| TNF- α, pg/mL, median (IQR) | 31.1 (81.0) | 30.7 (22.7) | 0.763 |
| Oxidized-LDL, ng/mL, median (IQR) | 114.7 (116.5) | 113.2 (122.6) | 0.946 |
| ESR, median (IQR) | 24.0 (22.0) | 34.0 (35.0) | 0.569 |
| Hemoglobin, median (IQR) | 12.7 (1.6) | 13.9 (1.3) | <0.001 |
| Intestinal barrier integrity, serum |  |  |  |
| Occludin, pg/mL, median (IQR) | 38.8 (27.3) | 29.3 (20.9) | 0.261 |
| Claudin-1, pg/mL, median (IQR) | 27.8 (39.8) | 17.4 (29.3) | 0.197 |
| Zonulin, ng/mL, median (IQR) | 3.6 (5.3) | 3.7 (4.7) | 0.815 |
| LPS, µg/mL), median (IQR) | 109.0 (80.3) | 103.2 (110.2) | 0.700 |
| LBP, µg/mL, median (IQR) | 3.5 (4.0) | 2.9 (6.3) | 0.780 |
| Intestinal barrier integrity, feces |  |  |  |
| Occludin, pg/mL, median (IQR) | 51.4 (56.6) | 43.5 (54.6) | 0.740 |
| Claudin-1, pg/mL, median (IQR) | 58.2 (59.1) | 46.2 (61.2) | 0.301 |
| Zonulin, ng/mL, median (IQR) | 7.7 (6.6) | 4.3 (20.0) | 0.410 |
| *Adipokines* |  |  |  |
| Adiponectin, µg/mL, median (IQR) | 12.8 (2.0) | 12.6 (5.6) | 0.111 |
| Leptin, ng/mL, median (IQR) | 18.2 (13.7) | 7.0 (11.4) | 0.007 |
| Resistin, ng/mL, median (IQR) | 6.1 (4.0) | 6.6 (4.1) | 0.994 |
| Metabolism and growth |  |  |  |
| IGF-1, µg/mL, mean (SD) | 128.7 (18.4) | 109.0 (15.8) | 0.001 |
| DAS28-CRP response at 6 months |  |  |  |
| Remission, n (%) | 27 (47.4) | 6 (46.2) | 0.591 |
| Low activity, n (%) | 15 (26.3) | 3 (23.1) | 0.558 |
|  |  |  |  |
| EULAR response at 6 months |  |  | 0.552 |
| Good response, n (%) | 35 (61.4) | 6 (46.2) |  |
| Moderate response, n (%) | 15 (24.6) | 5 (38.5) |  |
| No response, n (%) | 8 (14.0) | 2 (15.4) |  |

*Abbreviations: RA: rheumatoid arthritis; TNF-α: tumor necrosis factor alpha; SD: standard deviation; IQR: interquartile range; BMI: body mass index; RF: rheumatoid factor; ACPA: anti–citrullinated peptide antibody; DAS28-CRP: 28-joint Disease Activity Score based on C-reactive protein; HAQ: Health Assessment Questionnaire; CRP: C-reactive protein; IL-6: interleukin 6; IL-1β: interleukin 1 beta; LDL: low-density lipoprotein; ESR: erythrocyte sedimentation rate; LPS: lipopolysaccharide; LBP: LPS-binding protein; IGF-1: insulin-like growth factor 1; EULAR: European League Against Rheumatism.*
